# The prevalence and causes of visual impairment among children in Kenya – the Kenya eye study

**DOI:** 10.1186/s12886-020-01665-w

**Published:** 2020-10-07

**Authors:** Shadrack Muma, Stephen Obonyo

**Affiliations:** 1 Department of Public Health, Maseno University, Po Box 811, Kisumu, Kenya; 2 Department of Computing and Informatics, Strathmore University, Nairobi City, Kenya

**Keywords:** Kenya, Prevalence, Ocular diseases, Visual impairment, Refractive error

## Abstract

**Background:**

Visual impairment is the partial or complete loss of vision in which the presenting visual acuity lie between 6/18-no perceptions of light. In Kenya, little attention has been directed towards children vision and causes of visual impairment. Therefore, this study was designed to investigate the prevalence and causes of visual impairment in the children population of Kenya.

**Methods:**

This cross-sectional population-based study included 3400 (1800, 52.9% female) randomly selected children with a mean age of 12 ± 2 years (range 5–16 years). Visual acuity was taken using Snellens chart at 6 m. Anterior and posterior segment was assessed using slit lamp and indirect ophthalmoscope. The World Health Organization definition formed the baseline for calculating the mean prevalence of visual impairment.

**Results:**

Visual acuity measurements were available for 3240 (95.3%) participants. The mean prevalence of visual impairment based on pin-hole value was 1.7 ± 0.3% using World Health Organization definition. The prevalence of visual impairment based on presenting visual acuity value was 2.4 ± 0.7% using the World Health Organization definition. Multivariate analysis demonstrated that the presence of visual impairment on pin-hole increased significantly with increasing age (odds ratio 1.230, *P* = .021) and uncorrected refractive error (odds ratio 0.834, *P* = .032) according to World Health Organization definition. Cases of uncorrected refractive error remained the major cause for presenting visual impairment. Causes of visual impairment due to presenting visual acuity were nystagmus (14%), amblyopia (24%) and uncorrected refractive error (62%).

**Conclusion:**

The prevalence of visual impairment in Kenya is associated with age. Uncorrected refractive error remains the major causes of visual impairment.

## Background

Visual impairment is the partial or complete loss of vision and it is classified as moderate with presenting visual acuity of < 6/18–6/60, severe < 6/60–3/60, blind < 3/60-no perception of light. Visual impairment is considered a major issue of public health concern. There are raising cases of visual impairment and the World Health Organization gives a rough estimate of 1 billion people being visually impaired globally [1]. In Kenya about 0.7% of all rural Kenyans are blind in their better eye, and another 2.5% have vision which is substantially impaired [2]. The recent study conducted in Kenya, Nakuru County, estimated that 92,000 adults aged ≥50 years had visual impairment of whom 11,600 were blind, out of a total population of approximately 4.3 million [3]. The study focused on the adult population with little attention on the children population, hence there is need for studies on children population. Overally, visual impairment is ranked sixth in the global burden of disease in terms of disability-adjusted life-years and is associated with increased mortality [4–6]. In as much as there is a reduction in the prevalence of blindness in Sub-Saharan Africa [7], the numbers with visual impairment are rising as a result of increase in the population. However, data on prevalence of visual impairment among children population in Kenya has not been established.

Visual impairment is attributed to uncorrected refractive error, cataract, age-related macular degeneration, glaucoma, diabetic retinopathy, corneal opacity and trachoma [8]. However, the major leading cause of visual impairment is uncorrected refractive error which is manageable. Studies on causes of visual impairment have been conducted worldwide in high-income settings and only among the adults [9–12]. A study conducted in Kenya among adult population found that uncorrected refractive error was the main cause of visual impairment [13]. However, data from high-income groups is not applicable to low-income settings, and for effective planning, data from low income countries is required. Kenya being a developing country, the population increases daily with an estimated total fertility rate of 3.5 and a life expectancy of 67.5 years. Therefore, a lot of interest should be directed towards the younger generation so as to enhance good quality of life. However, there is paucity of data on causes of visual impairment in Kenya among the children population.

The aim of this study was to determine the prevalence and causes of visual impairment in a cohort of children population in Kenya.

## Methods

This cross-sectional study was carried out from January 2018 to February 2019. Participants were randomly selected from the 47 county referral hospitals in Kenya in the 8 provinces. The provinces includes: Nyanza, Western, Central, Eastern, Rift Valley, North Eastern, Nairobi and Coast. The list of facilities where the participants were recruited from included; Homa bay county referral hospital, Kakamega county referral hospital, Kiambu county referral, Embu county referral, Kericho county referral, Kitui county referral, Kenyatta National Hospital and Coast general hospital. The participants belonged to the cohort of children who had visited the hospitals three times consecutively. The sample was obtained from list of patients who had been visiting the facility over some time till 2017. The participants were randomly selected from the eight county referral hospitals listed above. Only children who could be traced with a record from the hospital were included in the study. Eligibility was based on visiting the same facility frequently and having the Universal Health Coverage card registered in the facility. The reason for universal health coverage was because it makes access to health facility easy hence frequent visit in case of any health complication. At the same time, the universal health coverage only works in the county referral hospitals hence the study area was more applicable. Finally the county referral hospitals have well established ophthalmology centre’s which allow for comprehensive examination of the patients. We excluded children with psychiatric history due to lack of concentration during ocular examination. For the randomly selected participants, consent was sought from their parents and assent from the children. To ensure a good response rate a consistent contact follow up was adopted. Participants who agreed were given a prior call to inform them on the examination area. Constant reminders were sent to the participants on when the examination would be conducted. The recruitment of the participants lasted for 6 months, that is from January to June 2018.

After examination participants were given a bottle of soda and bread just as a form of appreciation for taking their time to participate in the study. Being that the facilities had consultant ophthalmologists, participants who required more attention were reviewed by the ophthalmologists. The team consisted of ninety four optometrists with one hundred research assistants. The ocular history was recorded by the research assistants using structured questionnaires in Kiswahili. The parent’s knowledge was sought on amblyopia which is the decreased vision due to abnormal visual development, nystagmus which is the involuntary eye movement and refractive error. Immediately the participants arrived at the examination area, the history was taken followed by visual acuity. The visual acuity was recorded at 6 m using the Snellens chart. A presenting visual acuity which is the visual acuity in the better eye and visual acuity of 6/60 was considered severe, worse than 6/18 considered moderate. For participants who could not see 6/18, a pin hole was used to confirm if it’s a pathology or refractive error. For participants whose visual acuity improved on pin-hole, retinoscopy was done to determine the magnitude and type of refractive error they had. A subjective refraction was done to confirm the objective refraction. After retinoscopy, slit lamp assessment was done to examine the anterior segment for any abnormality. The pupil was dilated using tropicamide 0.5% to assess the posterior part of the eye. Hruby lens of +90D was used to assess the fundus. To allow for comparison with the World Health Organization, the definition of visual impairment was used [14].

Statistical analysis was carried using Statistical Package for Social Sciences software (SPSS, version 17.0). Descriptive statistics was conducted which included the mean, standard deviation (SD), median and percentages. The prevalence of visual impairment was determined based on age and gender. A chi-square test was conducted to compare the prevalence of visual impairment between different gender and age groups. Logistic regression analysis was conducted to compare associations of visual impairment.

## Results

A total of 3240 (1600, 49.4% male; from 8 county referral hospitals in the 8 provinces) out of 3400 eligible subjects (response rate: 95.3%) participated in the survey from January 2018 to February 2019 Table 1. All participants were Kenyans, with a mean age of 12 ± 2 years (range 5–16 years). Visual acuity measurements were available for 3181 out of 3240 (98.2%) subjects. Those participants who had no visual acuity score were significantly different from those with visual acuity score based on gender (49.4% vs. 50.6%, *P* = .014) and age (5 ± 4.3 vs. 11 ± 4.7, *P* = .012).

**Table 1:** Demographic characteristic of the respondents

Variables	Health Facility	Frequency	Percentage
Age group			
5–8		1160	35.8
9–12		1420	43.82
13–16		660	20.37
**Provinces**			
Nyanza	Homa bay	340	10.49
Western	Kakamega	420	12.96
Rift valley	Kericho	500	15.43
North eastern	Kitui	440	13.58
Nairobi	Kenyatta	600	18.52
Eastern	Embu	450	13.89
Central	Kiambu	290	8.95
Coast	Coast	200	6.17

The mean for presenting visual acuity was 0.12 ± 0.15 (range 6/36 to 6/9) Snellens chart. Based on best corrected visual acuity, the prevalence of visual impairment was 6/6 ± 0.2% using World Health Organization definition. The prevalence of visual impairment in relation to best corrected visual acuity reduced significantly with gender using the World Health Organization definition (*P* = .46), and age (5–8 (2.4%) versus 13–16 (0.5%) using the World Health Organization definition, *P* = .013). (Table 2).

**Table 2:** Prevalence of visual impairment according to presenting visual acuity in Kenya

	Male		Female	
	No visual impairment	Visual impairment	No visual impairment	Visual impairment
	WHO Criteria: ≤ 6/18			
	Count (%)	Count (%)	Count (%)	Count (%)
5–8	1054 (31)	81 (2.4)	168 (4.9)	123 (3.6)
9–12	408 (12)	54 (1.6)	209 (6.1)	342 (10.1)
13–16	646 (19)	17 (0.5)	103 (3.0)	564 (16.5)

The mean presenting visual acuity was (0.18 ± 0.26 (2.4 ± 0.7) using Snellens chart. Based on World Health Organization definition of pin-hole, the prevalence of visual impairment was 1.7 ± 0.3%. The prevalence of visual impairment based on presenting visual acuity reduced significantly with gender using World Health Organization criteria (*P* < .017). There was a significant difference on the prevalence of presenting visual acuity between men and women (49.4% versus 50.2% using World Health Organization criteria, *P* = .024). A bi-variate analysis showed that there was a statistically significant difference on presenting visual acuity and age (*P* = 0.02).

**Fig. 1:**
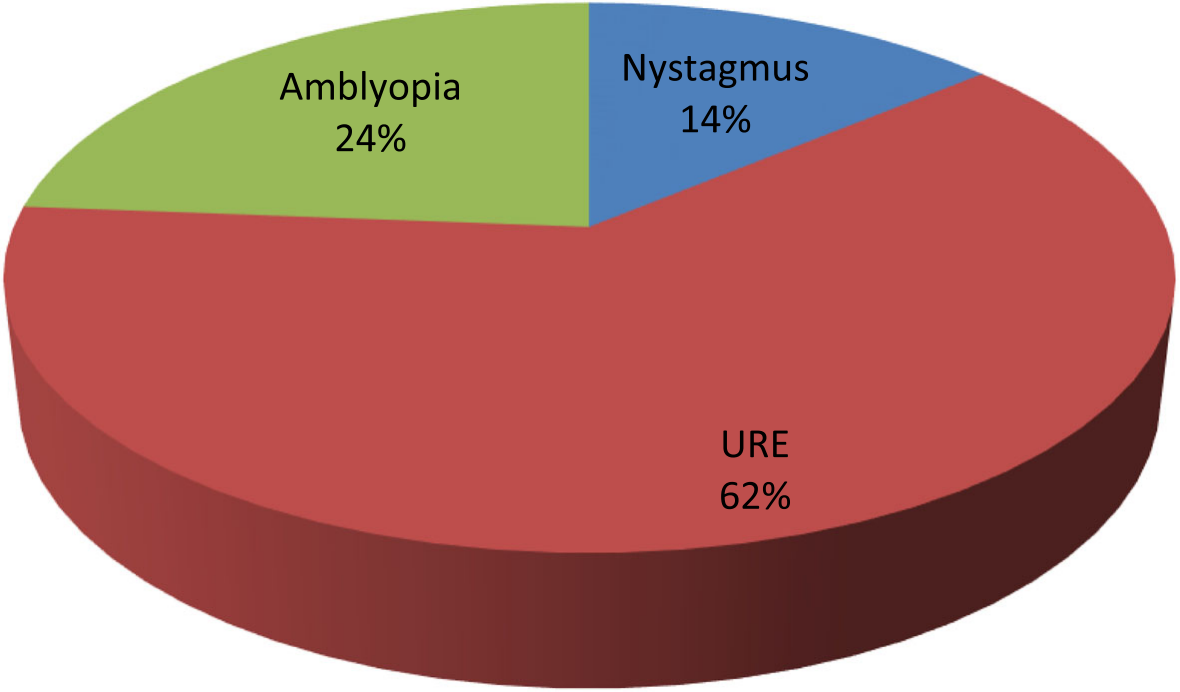
The causes of visual impairment among children in Kenya. Figure legend: the figure above shows the major causes of visual impairment among children in kenya. Abbreviation; URE uncorrected refractive error

Uncorrected refractive error (62%) was the main cause of presenting visual acuity impairment among children. Based on pin-hole visual acuity, nystagmus (14%), amblyopia (24%) and uncorrected refractive error (62%) were the main causes of visual impairment (Fig. 1). Visual impairment was significantly associated with uncorrected refractive error (*P* = 0.015). There were only two children aged 6 and 9 who had bilateral blindness.

## Discussion

In summary, Kenya with a population of 47 million the prevalence of visual impairment was 2.4% among the children population in Kenya. The 8 county referral hospitals involved in the study had relatively different demographic characteristics specifically on socio-economic activities. However, uncorrected refractive error remained the major cause of visual impairment with age being associated with visual impairment. To ensure cases of visual impairment as a result of uncorrected refractive error are curbed, early ocular examination and optical correction is desired. Most parents had little knowledge on the conditions associated with visual impairment. The conditions cuts across all age group, however on the basis of diagnosis they remain more significant among children. There was no significant association between visual impairment and nystagmus.

In comparison to other studies in other regions, the prevalence of visual impairment among children aged 5–16 years in Kenya was relatively lower [15–17]. This is attributed to the improved health care system in Kenya in the last 10 years. The Ophthalmic Division in Kenya has improved the health care and enhanced public awareness on ocular conditions such as refractive error. This has aided in early detection of ocular diseases and management making patients to regain their sight at an early age. We did not only assess the prevalence of visual impairment in children aged 5–16 years, but we also investigated the causes of visual impairment among the same children. The age group has not been investigated in developing countries due to low reports from children on ocular related complications [18]. The prevalence of visual impairment in our study was 2.4% based on presenting visual acuity in relation to World Health Organization criterion. The core reason for the presenting visual impairment in this age group was due to inadequate trained pediatrics eye care providers to conduct comprehensive eye examination 76%. Therefore there is a need for correction of refractive error among the children population.

The current finding is consistent with previous studies where uncorrected refractive error is the major cause of presenting visual acuity impairment. In a review of 137 studies of 78,543 participants from 82 countries, uncorrected refractive error remained the core leading causes of presenting visual impairment [19–21]. Being that refractive error is correctable; it is unfortunate that it still remains a major cause of visual impairment in Kenya even with the increase in eye care providers. In east Africa, Kenya is considered to be relatively advanced and awareness on refractive error through heath care professionals to the public should not be a barrier. Comprehensive eye assessment is necessary as it will determine whether spectacles are required. Future studies should address the solution to this problem. Apart from uncorrected refractive error, condition such as amblyopia contributes to visual impairment in Kenya.

The main limitation of the study was conducting a dry retinoscopy which could have resulted to over or under estimation of refractive error. Due to accommodation in children, the refractive error score obtained without dilation of the children makes the concentration reduces hence influencing the refractive error outcome. Comparing the study results with the World Health Organization classification was a major strength. This is because it makes the study to give a true map of the epidemiology of visual impairment in the region as a standard method is applied.

## Conclusion

Uncorrected refractive error still remains the major cause of visual impairment among children population in Kenya. In as much as the prevalence is not that high, a lot of attention should be directed towards child eye health to reduce incidences of visual impairment. To ensure that vision for the population is improved, eye care providers should do a comprehensive eye examination and dispense a pair of glass if need be. There is a need to increase the training of eye care providers in Kenya on pediatric eye care.

## Data Availability

The dataset generated and analyzed during the current study are not publicly available due to confidentiality issues as this is a study involving human beings but are available from the corresponding author on reasonable request.
